# Structural Properties and Sensing Performance of CeY_x_O_y_ Sensing Films for Electrolyte–Insulator–Semiconductor pH Sensors

**DOI:** 10.1038/s41598-017-03209-7

**Published:** 2017-06-07

**Authors:** Tung-Ming Pan, Chih-Wei Wang, Ching-Yi Chen

**Affiliations:** 1 Department of Electronics Engineering, Chang Gung University, Taoyuan, 33302 Taiwan; 2 Division of Urology, Chang Gung Memorial Hospital, Taoyuan, 33302 Taiwan

## Abstract

In this study we developed CeY_*x*_O_*y*_ sensing membranes displaying super-Nernstian pH-sensitivity for use in electrolyte–insulator–semiconductor (EIS) pH sensors. We examined the effect of thermal annealing on the structural properties and sensing characteristics of the CeY_*x*_O_*y*_ sensing membranes deposited through reactive co-sputtering onto Si substrates. X-ray diffraction, atomic force microscopy, and X-ray photoelectron spectroscopy revealed the structural, morphological, and chemical features, respectively, of the CeY_*x*_O_*y*_ films after their annealing at 600–900 °C. Among the tested systems, the CeY_*x*_O_*y*_ EIS device prepared with annealing at 800 °C exhibited the highest sensitivity (78.15 mV/pH), the lowest hysteresis voltage (1.4 mV), and the lowest drift rate (0.85 mV/h). Presumably, these annealing conditions optimized the stoichiometry of (CeY)O_2_ in the film and its surface roughness while suppressing silicate formation at the CeY_*x*_O_*y*_–Si interface. We attribute the super-Nernstian pH-sensitivity to the incorporation of Y ions in the Ce framework, thereby decreasing the oxidation state Ce (Ce^4+^ → Ce^3+^) and resulting in less than one electron transferred per proton in the redox reaction.

## Introduction

Due to increasing demands in environmental protection, health care and bio-nano technology, chemical- and bio-sensors based on semiconductors attract much attention as they can be miniaturized and integrated into small size electronic equipment for real time detection with high sensitivity. A dramatic change in the miniaturization of ion-selective membrane technology occurred when Bergveld demonstrated an ion-sensitive field-effect transistor (ISFET) by modifying a metal-oxide-semiconductor field-effect transistor (MOSFET) to expose the gate oxide film to an electrolyte solution^1, 2^. The theoretical chemical response of an ISFET sensor is the same as that of an ion-selective electrode. This sensor is replaced the fragile glass electrode with various gate oxides to detect a variety of ions. Since then, ISFETs offered several major advantages, including small size, rapid response, low output impedance, multiple ion sensor capability, and high compatibility for integration with complementary metal-oxide-semiconductor (CMOS) systems^3, 4^. Apart from ISFET, electrolyte-insulator-semiconductor (EIS) sensors without source and drain electrodes have relatively simpler structures and are easier to fabricate than ISFET devices. It is based on a MOSFET device without a metal gate for direct immersion in a buffer solution, the gate insulator material is a critical part of an ISFET or EIS device. The first sensitive pH response was attained with a SiO_2_ membrane. Later, several metal oxide materials (e.g. Al_2_O_3_, Ta_2_O_5_, SnO_2_, WO_3_^5–9^) were used as sensing membranes because these materials possess large surface areas and good sensing performance. In addition to conventional metal oxides, a number of refractory oxides having high-dielectric constants (high-κ), such as TiO_2_, ZrO_2_, HfO_2_, and Y_2_O_3_^10–12^, have been received considerable attention as pH-sensitive membranes for promising applications in ISFET and EIS devices due to their good sensing properties. Nevertheless, their structures are generally unstable and very reactive for long-term and higher temperature applications as a result of the formation of a interfacial layer between the high-k metal oxide and the Si substrate in their manufacture. The interface layer can affect not only the performance an EIS sensor but also the sensor inoperable. Consequently, it is needed to find new materials and alternative processes to diminish interfacial defects.

Rare earth (RE) oxide films are possible replacements for traditional SiO_2_ as the gate dielectric in advanced CMOS devices because of their large values of κ lower leakage currents, high resistivities, and good thermal stabilities^13–15^. We have previously reported the structural properties and sensing characteristics of several RE oxide films (e.g., Pr_2_O_3_, Nd_2_O_3_, Sm_2_O_3_, Er_2_O_3_) that behave as sensing membranes^16–19^. Among them, CeO_2_ thin films have several advantages, including strong adhesion, high refractive index, good redox, high mechanical strength, excellent thermal stability, for applications in humidity sensors, fuel cells, and gas sensors^20–22^. Ansari *et al*.^23^ also demonstrated that the nanostructured CeO_2_ film was deposited on Au electrode using the sol-gel method for glucose sensor. Besides the above properties, they possess a high dielectric constant, a small lattice mismatch with silicon, and good thermodynamic stability in contact with silicon, making them particularly attractive materials for use as alternative gate dielectrics in nano-CMOS, flash memory, and EIS devices^24–26^. The major concern when using RE oxide films as sensing membranes, however, is prone to moisture absorption due to their hygroscopic nature, which degrades permittivity through the formation of low-permittivity hydroxides. The moisture absorption of RE oxide films stems from the presence of oxygen vacancies in the films^27^. To address this issue, several researchers in literatures^27, 28^ have reported the incorporation of other elements (e.g., Ti, TiO_*x*_, Y) into RE dielectric films which suppress the moisture absorption of the RE oxide. In this concern, we have reported Ti-doped RE oxide sensing membranes in our earlier works^29–31^ which addresses the high hygroscopic nature of the RE oxide. The structural and sensing properties of CeY_*x*_O_*y*_ films as sensing membranes for EIS pH sensors have yet to be studied. In this work, we propose a CeY_*x*_O_*y*_ sensing film displaying super-Nernstian pH-sensitivity for use in EIS pH sensors. We have investigated the effects of thermal annealing on the structural properties and sensing characteristics of CeY_*x*_O_*y*_ thin films deposited on Si substrates through reactive co-sputtering. Moreover, we have employed X-ray diffraction (XRD), X-ray photoelectron spectroscopy (XPS), and atomic force microscopy (AFM) to monitor the quality of the CeY_*x*_O_*y*_ sensing films and determine the optimal annealing conditions. We measured the sensing characteristics (pH sensitivity, hysteresis, and drift) of these CeY_*x*_O_*y*_ films. The sensitivity of one such CeY_*x*_O_*y*_ EIS sensor (78.15 mV/pH) was higher than that expected from the Nernst law (59.2 mV/pH). We attribute this super-Nernstian response to the incorporation of Y ions into the Ce framework, thereby decreasing the oxidation state of Ce (Ce^4+^ → Ce^3+^) and forming a mixed state of oxidized and reduced CeO_*x*_ in the solution; as a result, less than one electron per proton would be transferred in the redox reaction.

## Methods

### Fabrication

**Figure 1:**
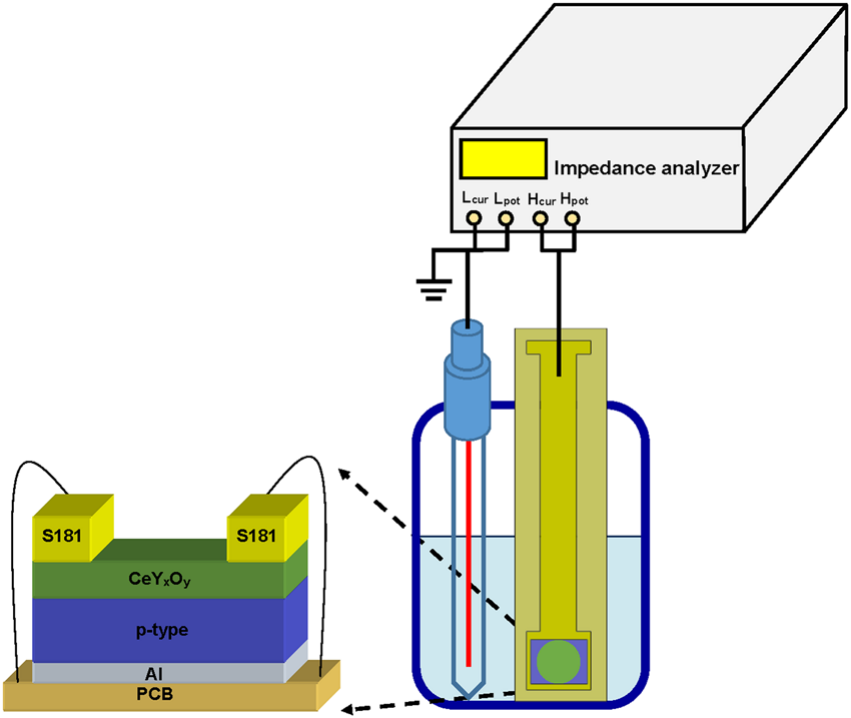
Schematic representation of the structure of a CeY_*x*_O_*y*_ EIS sensor.

EIS device structures incorporating CeY_*x*_O_*y*_ sensing films were fabricated on 4-in p-type (100) Si wafers. Prior to deposition of the CeY_*x*_O_*y*_ film, the wafer was cleaned through standard RCA cleaning and then treated with 1% HF to remove the native oxide. Subsequently, ~60 nm CeY_*x*_O_*y*_ film was deposited on the Si substrate through rf co-sputtering from cerium oxide and yttrium targets with a mixture of Ar and O_2_ (Ar:O_2_ = 25:5) ambient during sputtering. During the sputtering of CeY_*x*_O_*y*_ film, the rf power of CeO_2_ and Y targets was 150 and 150 W, respectively. The chamber pressure was maintained at ~10^−2^ Torr and the deposition rate of the film was ~1.5 nm/min. The samples were subjected to rapid thermal annealing (RTA) in O_2_ ambient for 30 s at 600, 700, 800, or 900 °C to achieve a crystalline (CeY)O_2_ film. To form a backside contact, the backside SiO_2_ layer was removed in a buffer oxide etchant (BOE) and then an Al film (400 nm) was deposited using a thermal evaporator. To define the sensing area of the deposited CeY_*x*_O_*y*_, an automatic robotic dispenser was used with an adhesive silicone gel (S181) acting as a segregating layer. The EIS device was assembled on a Cu wire on a custom-made printed circuit board by silver glue. To avoid leakage of the electrolyte, adhesive epoxy was encapsulated between EIS film and the Cu. The structure of the fabricated CeY_*x*_O_*y*_ EIS device is illustrated in Fig. 1.

### Characterization

After RTA, the structural and compositional properties of the CeY_*x*_O_*y*_ sensing films were investigated using a combination of XRD and XPS. The XRD patterns were recorded on a Bruker D8 discover diffractometer with Cu K_α_ radiation (λ = 0.1541 nm) in a scanning angle (2θ) range of 20–60° at a scanning speed of 1° min^−1^, operated at 40 kV and 40 mA, respectively. XPS was conducted using a Physical Electronics Quantum 2000 scanning ESCA microprobe, with a focused monochromatic Al K_α_ X-ray (1486.7 eV) source for excitation and a spherical section analyzer. Curve fitting of XPS spectra was performed using a Shirley background and a nonlinear least-squares curve-fitting program with a Gaussian/Lorentzian product function. The Gaussian/Lorentzian mixture was set at 0.5. The binding energies were calibrated by setting the value for the C 1 s orbital to 285 eV; they are accurate to within ± 0.2 eV. AFM images of the surfaces of the CeY_*x*_O_*y*_ films were recorded in air using a Solver P47 instrument (Russian NT-MDT), with a silicon probe operated in tapping mode. Surface roughnesses were determined from images obtained over an area of 3 × 3 μm^2^.

### Measurement

The pH-sensitivity of the CeY_*x*_O_*y*_ sensing films was determined by measuring the capacitance–voltage (*C*–*V*) curves of their EIS devices against pH buffer solutions (Merck), using a Ag/AgCl reference electrode and a Hewlett–Packard (HP) 4284 A LCR meter (ac signal frequency: 500 Hz); to avoid interference from light and noise, each experiment was performed in the dark at room temperature.

## Results and Discussion

### Structural Properties

**Figure 2:**
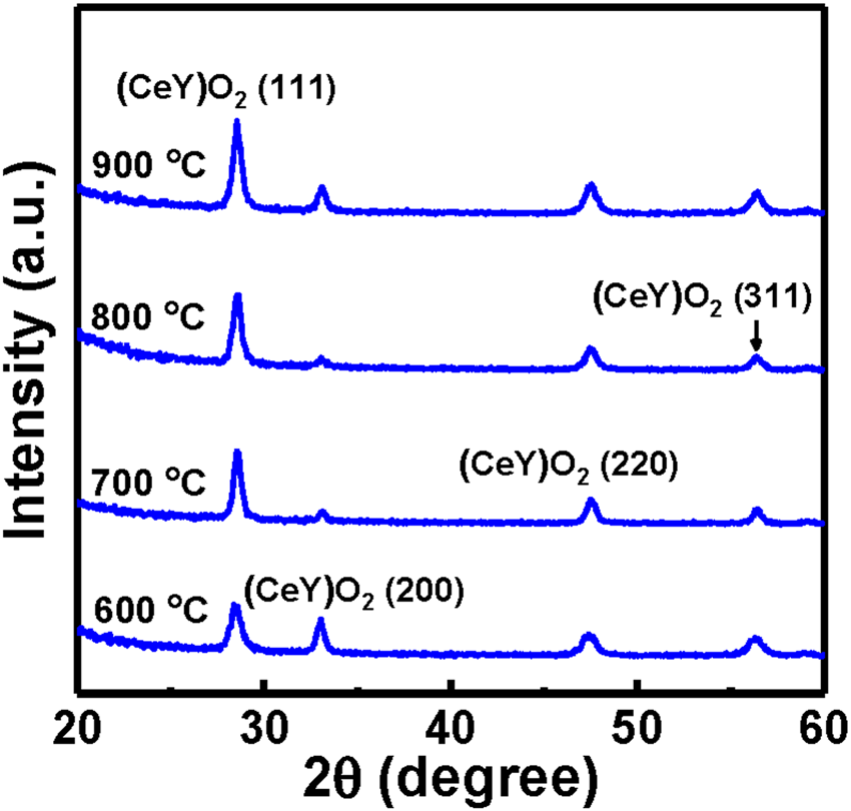
XRD patterns of CeY_*x*_O_*y*_ films that had been annealed at various temperatures.

In order to explore the microstructure of the annealed CeY_*x*_O_*y*_ sensing films, XRD measurements were carried out. It was found that the sharpness, intensity, and orientation of the diffraction peaks were sensitive to the thermal annealing condition. In Fig. 2, we show the XRD patterns of CeY_*x*_O_*y*_ sensing films for four different annealing temperatures, namely, 600 °C, 700 °C, 800 °C and 900 °C. Changes in the XRD patterns of the samples due to the thermal annealing can be seen clearly in Fig. 2. The diffraction peaks of all the films assigned to the (111), (200), (220), and (311) planes are indexed to the face-centered cubic structure of (CeY)O_2_ crystals. The XRD pattern of the film annealed at 600 °C featured a strong (CeY)O_2_ (111) peak, (CeY)O_2_ (200) peak, and two weak (CeY)O_2_ (220) and (311) peaks. RTA improved the crystalline structure of the (CeY)O_2_ films. The intensity of the (CeY)O_2_ (111) peak increased upon increasing the RTA temperature, consistent with a more crystalline CeY_*x*_O_*y*_ structure. A strong (CeY)O_2_ (111) peak, (CeY)O_2_ (200) peak, and two weak (CeY)O_2_ (220) and (311) peaks appeared in the patterns of the samples that had been annealed at 800 and 900 °C, suggesting the formation of a stoichiometric (CeY)O_2_ film. We attribute this annealing behavior to the Y atoms reacting with CeO_*x*_ to provide a more stoichiometric CeY_*x*_O_*y*_ structure.

**Figure 3:**
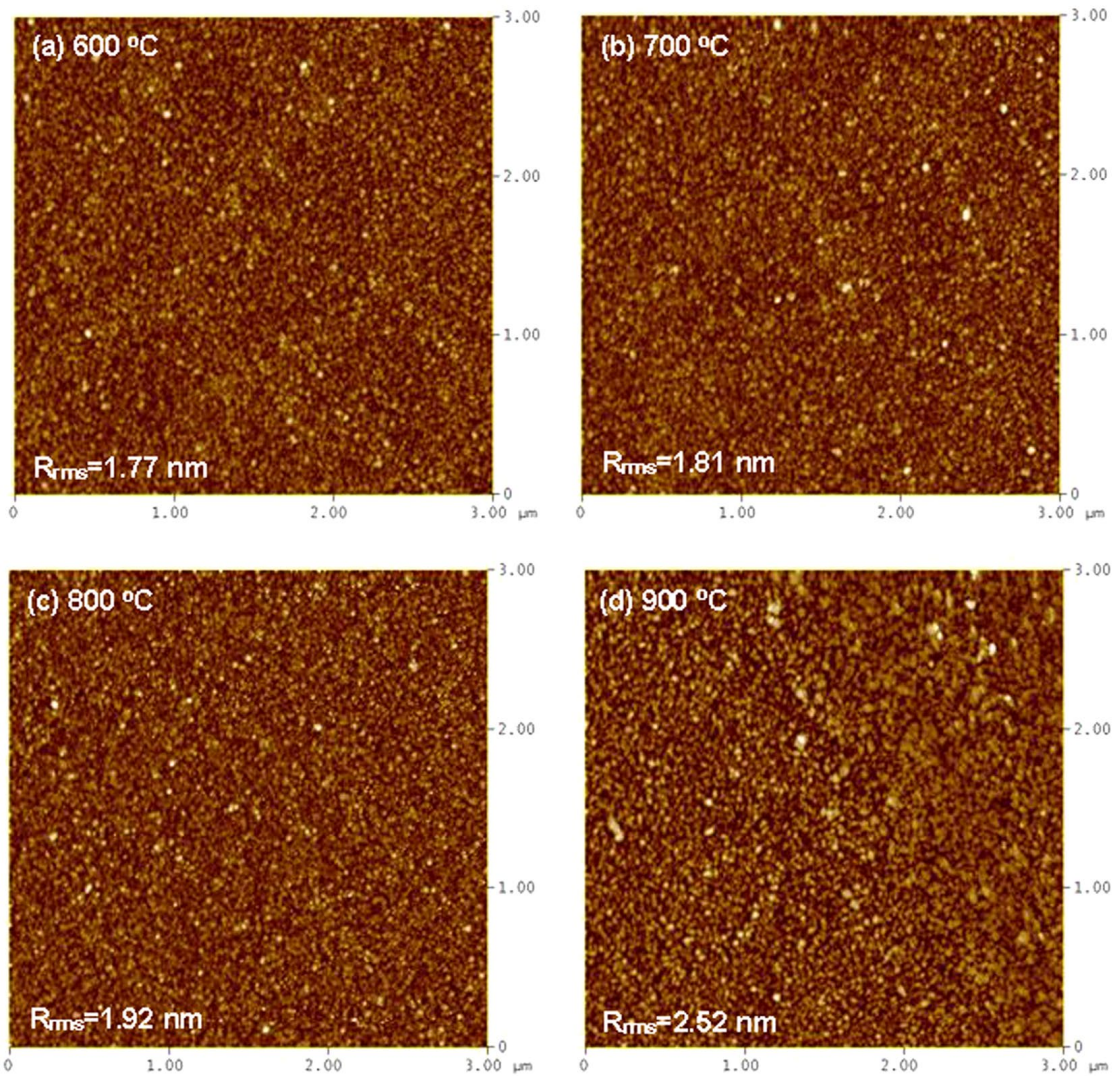
AFM surface images of CeY_*x*_O_*y*_ sensing films that had been annealed at (**a**) 600, (**b**) 700, (**c**) 800, and (**d**) 900 °C.

The morphology of the CeY_*x*_O_*y*_ films prepared at various temperatures is revealed by AFM images. Figure 3 displays AFM surface images of CeY_*x*_O_*y*_ films that had been annealed at different temperatures. The surface roughness (rms) values of 1.77, 1.81, 1.92, and 252 nm were determined for the CeY_*x*_O_*y*_ films treated at 600, 700, 800, and 900 °C, respectively. It can be see that the surface roughness value increased with increasing the annealing temperature. This behavior is attributed to the aggregation of the native grains into the larger clusters upon annealing^32^. This different cluster size affects the surface morphology of the films. The surface roughnesses of the CeY_*x*_O_*y*_ films annealed at the various RTA temperatures were consistent with the intensities of the diffraction peaks in Fig. 2; therefore, the AFM and XRD data are in good agreement. Figure 3(d) depicts that the CeY_*x*_O_*y*_ film annealed at 900 °C had larger clusters and becomes rougher. We suspect that self-diffusion of Ce and Y atoms increased during high-temperature annealing, enhancing the clustering of grains and, thereby, increasing the surface roughness of the CeY_*x*_O_*y*_ film. An increase in grain size might improve the sensing performance when the film was used for the membrane. In contrast, Fig. 3(a) demonstrates that the CeY_*x*_O_*y*_ film at 600 °C featured smaller grains and showed smoother surface.

**Figure 4:**
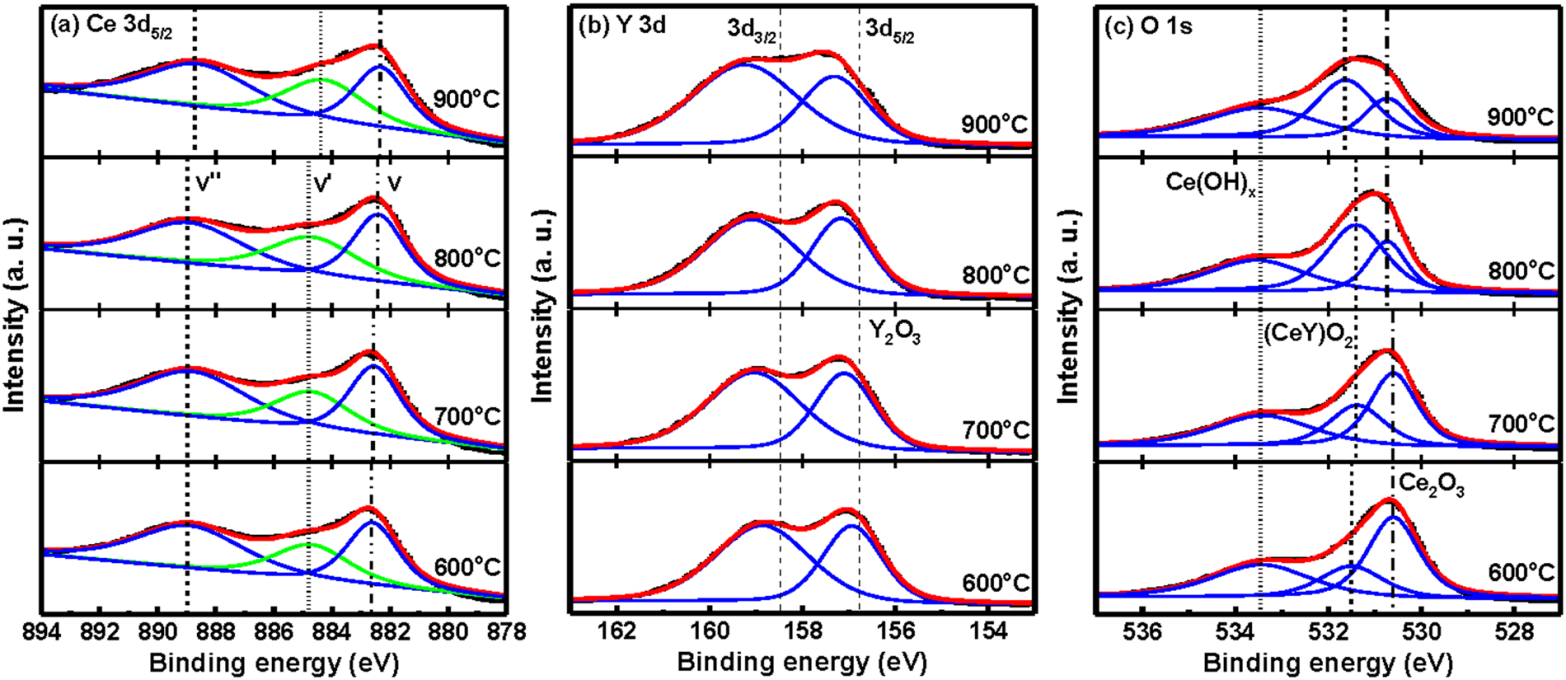
XPS spectra displaying the (**a**) Ce 3d, (**b**) Y 3d, and (**c**) O 1 s energy levels in CeY_*x*_O_*y*_ films that had been annealed at various temperatures.

To further investigate the surface composition and chemical states, the CeY_*x*_O_*y*_ films after RTA at various temperatures were performed by XPS technique. The Ce 3d, Y 3d, and O 1 s spectra of the CeY_*x*_O_*y*_ films prepared at different temperatures are shown in Fig. 4(a), (b) and (c), respectively. The Ce 3d_5/2_ spectra in Fig. 4(a) can be deconvoluted into three peaks: *v* (~882.6 eV), *v*′ (~884.7 eV) and *v*′′ (~888.9 eV). The 3d^1^°4f^0^ state of Ce^4+^ species are labeled as *v* and *v*′′, whereas the 3d^10^4f^1^ state of Ce^3+^ is represented as *v*′^33^. The binding energy of the Ce 3d_5/2_ peak at 882.1 eV^34^ for the CeO_2_ film is taken as energy reference, while that at 885.3 eV is assigned to the Ce_2_O_3_ structure^34^. Three *v*, *v*′ and *v*′′ peak binding energies exhibit quite similar evolutions, indicating no samples modification with depth. From Fig. 4, it is found that the main chemical valence of cerium for + 4 oxidation state and a small amount of + 3 oxidation state co-existed in the film. The effective ionic radii of Ce^4+^, Ce^3+^ and Y^3+^ are 0.97, 1.14 and 1.01 Å^35^, respectively. The + 3 oxidation state of cerium was introduced by the spontaneous transformation of smaller radius Ce^4+^ due to larger radius Ce^3+^, the addition of smaller radius Y^3+^ can compensate the lattice expansion induced by larger Ce^3+^ ions. A shift of the *v* peak position toward lower binding energy, from 882.6 to 882.3 eV, prepared upon increasing the RTA from 600 to 900 °C, indicative of reactions of the Ce and Y atoms with O atoms to form a stoichiometric (CeY)O_2_ structure. For the film annealed at 600, 700 and 800 °C, the *v*′ and *v*′′ peak positions of were fixed at 884.7 and 888.9 eV, but 900 °C-annealed sample suddenly shifted toward lower binding energy by 0.3 and 0.2 eV, respectively. It was thus interpreted that the deposited CeY_*x*_O_*y*_ film undergoes solid-phase intermixing with Si from the substrate during high-temperature annealing to form a Ce-silicate layer. The silicate layer is speculated to result from reactions between silicon and CeO_x_ formed at the surface of oxide film and Si substrate. The Y 3d_3/2_ and 3d_5/2_ double peaks of the Y_2_O_3_ reference appeared at 158.5 and 156.7 eV^36^, respectively. Figure 4(b) displays these signals for the film annealed at 600 °C, at 158.9 and 157 eV, respectively, suggesting a poor (CeY)O_2_ structure incorporating Y in the form of CeY(OH)_*x*_, which probably formed at the surface of the sample during its exposure to air. The positions of the Y 3d_3/2_ and 3d_5/2_ peaks shifted to higher binding energies upon increasing the annealing temperature. For the sample subjected to RTA at 800 °C, the binding energies of the Y 3d_3/2_ and 3d_5/2_ orbitals were 159.1 and 157.2 eV, respectively, reflecting the presence of (CeY)O_2_ structures. The intensities of the Y 3d_3/2_ and 3d_5/2_ split peaks provided by CeY_*x*_O_*y*_ remained quite constant for annealing temperatures of up to 800 °C, but the former increased suddenly while the latter decreased after annealing at 900 °C. This phenomenon is consistent with the reactions of Ce atoms with O and Si atoms to form a thicker Ce-silicate layer. Figure 4 (c) presents the O 1 s spectra for the annealed CeY_*x*_O_*y*_ films, with appropriate curve-fitting of the peaks. The O 1 s peak of the Ce_2_O_3_ reference appeared at 530.6 eV^37^. In the three spectra, the O 1 s peaks at 533.4, 531.5 (531.4 eV for 700 and 800 °C, 531.6 eV for 900 °C), and 530.6 eV (530.7 eV for 800 and 900 °C) represent the Ce–OH, Ce–O–Y, and Ce–O bonds, respectively. For 600 °C-annealed sample, the O 1 s peak intensities provided by Ce(OH)_x_ was larger compared to other annealing temperatures, respectively. This result may be attributed a poor CeY_x_O_y_ film to the hydroxide formation after exposure to an air ambient. The moisture absorption of RE oxide film may be attributed to the metal defects or oxygen vacancies in the films^38^. The intensity of the O 1 s peak corresponding to (CeY)O_2_ increased upon increasing the RTA temperature—except at 900 °C, where it decreased relative to the signal for Ce_2_O_3_. This behavior suggests that Ce and Y atoms reacted with O atoms to form a stoichiometric (CeY)O_2_ film. For the CeY_*x*_O_*y*_ component of the sample annealed at 900 °C, the O 1 s peak shifted to higher binding energy by 0.1 or 0.2 eV relative to those of the samples subjected to the other annealing temperatures. In addition, the intensity of the O 1 s peak for the Ce_2_O_3_ component of the sample annealed at 900 °C was weaker than other RTA temperatures. During high-temperature annealing, the Ce atoms diffused readily from the CeO_*x*_ film to form a thicker Ce-silicate layer at the CeY_*x*_O_*y*_–Si interface^39^.

### Sensing Characteristics

The metal gate of the MOSFET is replaced by a reference electrode, while the liquid in which this electrode is present makes contact with the gate oxide. In case of an ISFET the gate voltage is the voltage at the reference electrode, usually grounded reference electrode, but the threshold voltage comprises also terms which reflect the interfaces between the liquid and the gate oxide on the one side and the liquid and the reference electrode at the other side. The threshold voltage (*V*_th_) of an ISFET device is defined by the equation^40^

1 $${V}_{th}={E}_{ref}-\psi +{\chi }^{sol}-\frac{{{\rm{\Phi }}}_{Si}}{q}-(\frac{{Q}_{SS}+{Q}_{SC}}{{C}_{OX}}-2{\varphi }_{fp}).$$

where *E*_ref_ is the reference electrode potential relative to vacuum, *ψ* is the surface potential, which results from a chemical reaction, usually governed by the dissociation of oxide surface groups, *χ* ^sol^ is the surface dipole potential of the solution, *Φ*_Si_ is the Si work function, *q* is the elementary charge, *Q*_ss_ is the surface state density at the Si surface, *Q*_SC_ is the depletion charge in Si, *C*_ox_ is the gate oxide capacitance per unit area, and *ϕ*_fp_ is the Fermi potential of semiconductor (p-type for the n-channel MOSFET considered here). All terms usually refer to constant but does not include ψ, because this term makes the ISFET sensitive to the electrolyte pH, which is controlling the dissociation of the oxide surface groups. Designing a pH sensitive ISFET with a maximum sensitivity needs therefore a detailed study of the oxide-electrolyte interface to choose the best oxide film, which is not a priority for SiO_2_ in MOSFETs. In an ISFET device, the change in the surface potential at the oxide–aqueous electrolyte interface will shift the threshold voltage. It is mainly related to the charge concentration, which can be interpreted using the site-binding model to characterize the properties of an oxide–electrolyte interface^41^. The surface of metal oxide usually contains hydroxyl groups. These groups can donate or accept a proton from the solution, leaving a negatively charged or a positively charged surface group, respectively. The equilibrium reactions can occur between protons in the solution and the hydroxyl groups formed at the oxide-solution interface. The mechanism responsible for the oxide surface charge can be explained by the site-binding model, which describes the equilibrium between the so-called amphoteric Si-OH surface sites and the H^+^-ions in the solution. In the site-binding model, the terminal OH groups on the surface of the gate oxide can be neutral in the form (OH), protonated (OH_2_^+^; i.e., accepted a proton), or deprotonated (O^–^; i.e., donated a proton) at the oxide–electrolyte interface. At low pH (high proton concentration in solution), the equilibrium is shifted toward a protonated (i.e., positively charged) surface, which becomes a positive site. As a result, the *C*–*V* curve is shifted to the right direction. Around the point of zero charge, a general expression for the pH sensitivity of an ISFET sensor is given by^41^

2 $$\frac{d{\rm{\psi }}}{dpH}=2.303\frac{kT}{q}\frac{{\rm{\beta }}}{{\rm{\beta }}+1}$$

where *k* is Boltzmann’s constant, *T* is the absolute temperature, and *β* is a parameter that denotes the chemical sensitivity of the gate oxide and is proportional to the density of reactive sites, given by

3 $$\beta =\frac{2{q}^{2}{N}_{s}\sqrt{{K}_{a}{K}_{b}}}{kT{C}_{DL}}$$

where *N*_s_ is the total number of surface sites per unit area, *K*_a_ and *K*_b_ are the equilibrium constants at the acid and base point, respectively, and *C*_DL_ is the double-layer capacitance derived from the Gouy–Chapman–Stern model^42^. In this model, the double-layer capacitance consists of a series network of a Helmholtz-layer capacitance (the Stern capacitance) and a diffuse-layer capacitance. The Helmholtz layer model indicate that the ions in the solution have a finite size and the centers of the ions cannot approach the surface any closer compared with the ionic radius (including a possible water layer). The diffuse layer comprises the same amount of charge (of opposite sign) as the oxide surface charge, since the Helmholtz layer is by definition no containing any charge. If β parameter approaches infinite, the ISFET has a theoretical maximum value of accurately 59.6 mV/pH at 300 K, which is also called the maximum Nernstian sensitivity.

**Figure 5:**
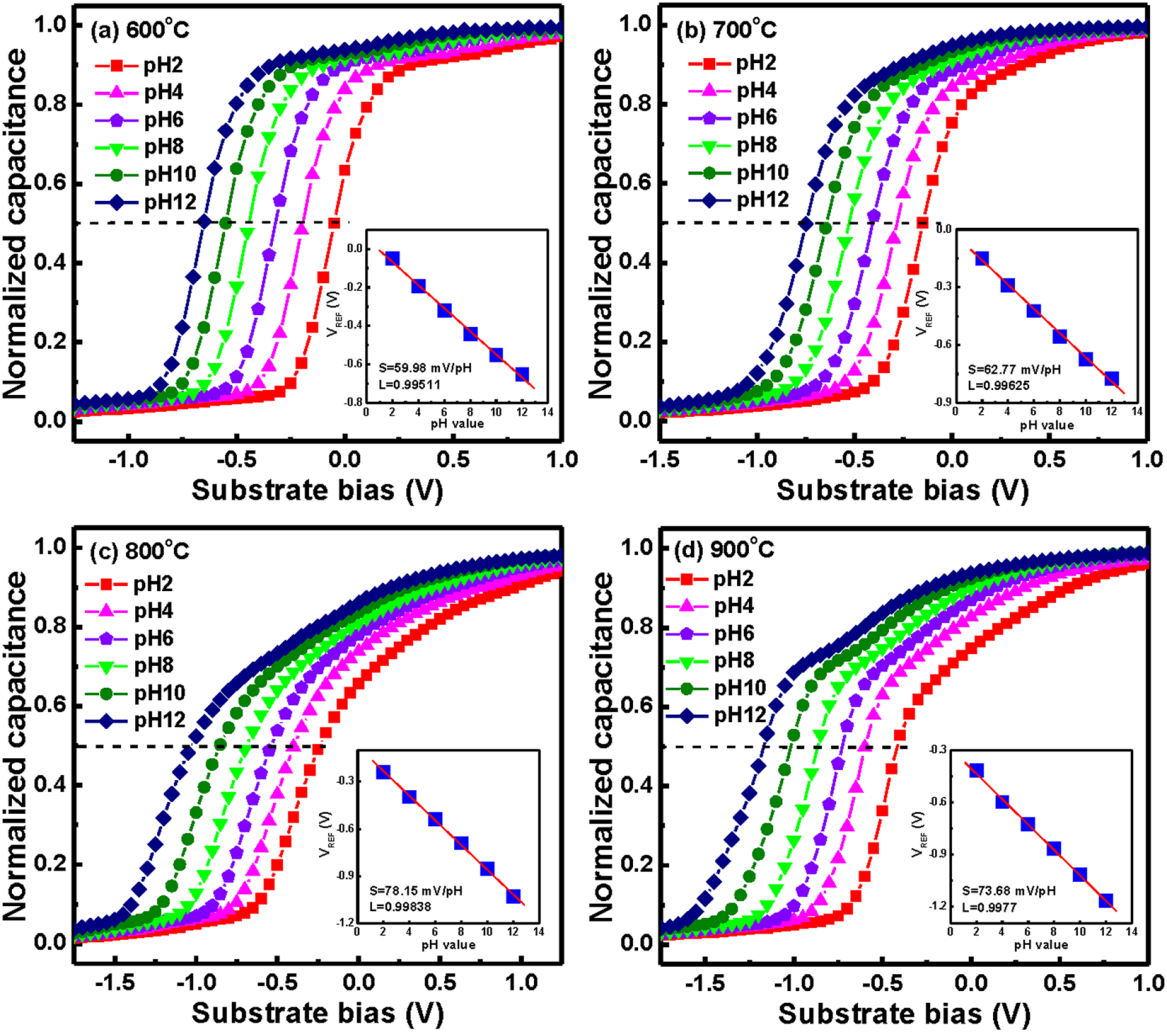
Responses of the *C*–*V* curves for CeY_*x*_O_*y*_ EIS sensors that had been prepared with annealing at (**a**) 600, (**b**) 700, (**c**) 800, and (**d**) 900 °C. Insets: Reference voltages plotted with respect to pH for the CeY_*x*_O_*y*_ EIS sensors.

To achieve the high sensitivity of an EIS sensor, we evaluated the sensing performance of the CeY_*x*_O_*y*_ EIS devices annealed at various temperatures. Figure 5(a–d) display the pH-dependence of a group of *C*–*V* curves for the CeY_*x*_O_*y*_ EIS devices prepared with annealing at 600, 700, 800, and 900 °C, respectively, and measured at values of pH in the range from 2 to 12. The change in the number of hydrogen ions on the sensing films led to shifts in the normalized *C*–*V* curves. We attribute the kinks in the *C*–*V* curves to the presence of interface traps between the CeY_*x*_O_*y*_ film and Si substrate^43^. Here, the reference voltage (*V*_REF_) for each pH buffer solution was the voltage required to obtain a normalized capacitance of 0.5. The insets to Fig. 5 (a–d) display the *V*_REF_ as function of pH values for the CeY_*x*_O_*y*_ EIS devices that had been prepared with the various annealing temperatures. The pH-sensitivities of the CeY_*x*_O_*y*_ films after RTA at 600, 700, 800, and 900 °C were determined to be 59.98, 62.77, 78.15, and 73.68 mV/pH, respectively. Thus, among these tested systems, the CeY_*x*_O_*y*_ EIS device annealed at 800 °C exhibited the highest sensitivity, possibly because of the higher surface roughness (AFM) of its CeY_*x*_O_*y*_ sensing film. A higher surface roughness will increase the surface site density and, therefore, improve the detection sensitivity, as previously discussed for the site-binding model. Nevertheless, the sensitivity of the CeY_*x*_O_*y*_ film annealed at 900 °C was lower than that of the film annealed at 800 °C, presumably because of a thicker silicate layer formed at the CeY_*x*_O_*y*_–substrate interface after treatment at such a high temperature^39^. Moreover, the pH-responses for the CeY_*x*_O_*y*_ EIS sensors were higher than that expected (59.6 mV/pH) from the Nernst law, possibly because the addition of Y ions into the Ce framework enhanced the decrease in the Ce oxidation state (Ce^4+^ → Ce^3+^). The wide application of ceria (CeO_2_) is driven by its ability to store and release oxygen (by creating oxygen vacancy sites) due to the easily accessible redox cycle of cerium ions (${{\rm{Ce}}}^{3+}\rightleftarrows {{\rm{Ce}}}^{4+}$)^44^.

During the oxygen release process, the volume of the Ce compound increases in proportion to the change in the Ce oxidation state from Ce^4+^ to Ce^3+^. The stress energy resulting from this volume increase would enhance any further valence change of the Ce oxidation state. The introduction of Y ions into the Ce framework would compensate for the increase in volume and ease the change in valence of the Ce oxidation state. The potential response with respect to pH is related to the equilibrium between the oxidized and reduced forms of CeO_*x*_. The hydroxyl groups can be considered as amphoteric sites, according to the site-binding theory, which can get or release a proton, for example:

4 $${\rm{C}}{\rm{e}}{\rm{O}}({\rm{O}}{\rm{H}})\leftrightarrows {{\rm{C}}{\rm{e}}{\rm{O}}{\rm{O}}}^{-}+{{\rm{H}}}^{+}$$

5 $${\rm{C}}{\rm{e}}{\rm{O}}({\rm{O}}{\rm{H}})+{{\rm{H}}}^{+}\leftrightarrows {{\rm{C}}{\rm{e}}{\rm{O}}({\rm{O}}{\rm{H}}}_{2}{)}^{+}$$

6 $${2{\rm{C}}{\rm{e}}({\rm{O}}{\rm{H}})}_{3}+{{\rm{H}}}_{2}{\rm{O}}\leftrightarrows {{\rm{C}}{\rm{e}}}_{2}{{\rm{O}}({\rm{O}}{\rm{H}})}_{6}+{2{\rm{H}}}^{+}+{2{\rm{e}}}^{-}$$

In Eqs (4–6), these reactions do not need electron transfer because the number of electrons transferred is equivalent to that of the protons (H^+^). As a result, the pH-sensitivity is estimated to be 59 mV/pH when considering the redox potential^45^. Nevertheless, pH sensitivities of over 59 mV/pH have been measured for other electrodes (e.g., IrO_x_, Ta_2_O_5_)^46, 47^. We suspect that a change in the Ce ion valence occurs during the reversible reaction from Ce^4+^ to Ce^3+^. The film can be considered only as an acidic oxyhydroxide, which the amphoteric character of the hydroxyl groups can be neglected, each state of the film with its own degree of proton dissociation:

7 $${{\rm{C}}{\rm{e}}({\rm{O}}{\rm{H}})}_{3}\leftrightarrows {{\rm{C}}{\rm{e}}({\rm{O}}{\rm{H}})}_{2}{{\rm{O}}}^{-}+{{\rm{H}}}^{+}$$

8 $${{\rm{C}}{\rm{e}}}_{2}{\rm{O}}{({\rm{O}}{\rm{H}})}_{6}\leftrightarrows {{\rm{C}}{\rm{e}}}_{2}{\rm{O}}{({\rm{O}}{\rm{H}})}_{3}{{{\rm{O}}}_{3}}^{3-}+3{{\rm{H}}}^{+}$$

Substitution of redox reactions (7) and (8) into reaction (9) yields

9 $${2{\rm{C}}{\rm{e}}({\rm{O}}{\rm{H}})}_{2}{{\rm{O}}}^{-}+{{\rm{H}}}_{2}{\rm{O}}\leftrightarrows {{\rm{C}}{\rm{e}}}_{2}{\rm{O}}{({\rm{O}}{\rm{H}})}_{3}{{{\rm{O}}}_{3}}^{3-}+3{{\rm{H}}}^{+}+2{{\rm{e}}}^{-}$$

For this reaction, only two electrons are transferred for every three protons; in this case, the maximum slope for a Nernst equation could reach as high as 88 mV/pH^48^. Thus, experimental observations of pH sensitivities of greater than 59 mV/pH might be explained by considering mixed oxidized and reduced CeO_*x*_ states that lead to less than one electron per proton being transferred in the redox reaction. In contrast, for pH-sensitivities of less than 59 mV/pH, more than one electron would be transferred per proton. In this study, our CeY_*x*_O_*y*_ films exhibited sensitivities to pH that are superior to those of sensing materials commonly used for ISFET and EIS sensors such as, Nb_2_O_5_ (52.12 mV/pH)^49^, Y_2_O_3_ (54.5 mV/pH)^12^, CeO_2_ (58.76 mV/pH)^26^, ZnO_2_ (58 mV/pH)^50^, Pr_2_O_3_ (52.9 mV/pH)^16^, Nd_2_O_3_ (56.01 mV/pH)^17^, Sm_2_O_3_ (56.2 mV/pH)^18^, Er_2_O_3_ (57.58 mV/pH)^19^, and Eu_2_Ti_2_O_7_ (59.03 mV/pH)^29^.

**Figure 6:**
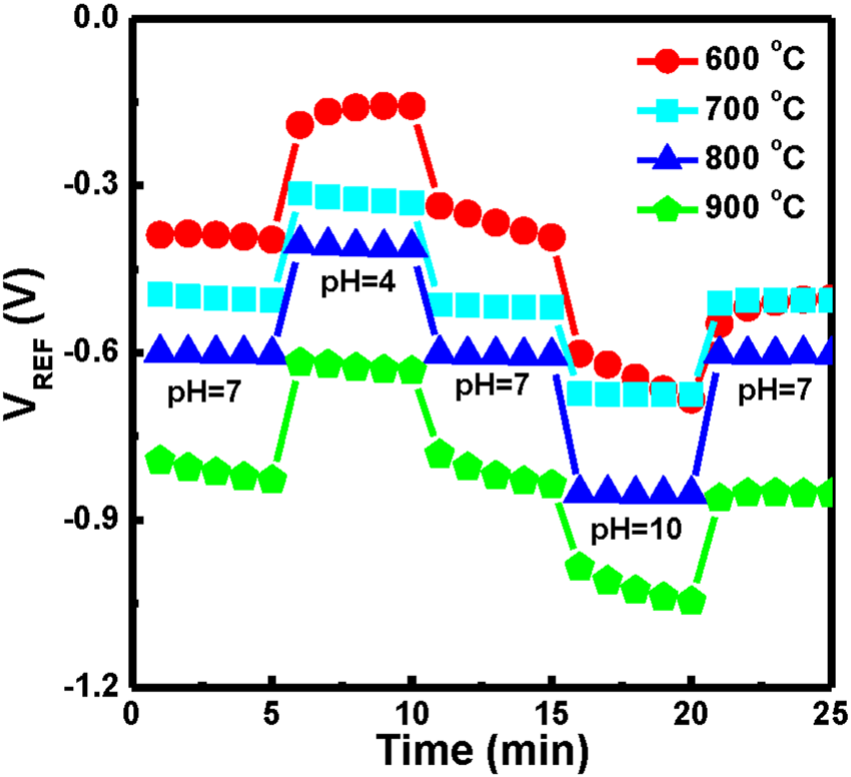
Hysteresis voltages during the pH loop 7→4→7→10→7 of CeY_*x*_O_*y*_ EIS devices that had been prepared with annealing at various temperatures.

The hysteresis and drift phenomena of a gate insulator influence the performance of ISFET and EIS sensors. A surface-site model can describe hysteresis characteristics when assuming that a fraction of the sites will respond slowly to changes in pH^5^ as a result of imperfections in the insulator film; these imperfections lead to porosity that produces interior sites that react with the ions in the solution. We tested our CeY_*x*_O_*y*_ EIS capacitors through a pH loop of 7→4→7→10→7 over a period of 25 min. We define the hysteresis voltage as the difference in reference voltage between the initial (first pH 7) and terminal (last pH 7) voltages in the pH loop. Figure 6 presents the hysteresis voltages of CeY_*x*_O_*y*_ EIS devices that had been prepared with annealing at various temperatures; they were immersed in buffer solutions having various values of pH in an alternating cycle (pH 7, pH 4, pH 7, pH 10, pH 7), for 5 min in each solution. The hysteresis voltage of the CeY_*x*_O_*y*_ EIS devices annealed at 600, 700, 800, and 900 °C were 126.3, 5.9, 1.4, and 40.9 mV, respectively. Thus, the EIS sensor annealed at 800 °C exhibited the smallest hysteresis voltage (1.4 mV), while that annealed at 600 °C had the highest (126.3 mV). Defects and vacancies in the films may have caused ions to attach to the surface and, thereby, delay the response to the reference voltage. Annealing at 800 °C could diminish the extent of crystal defects and, thereby, minimize the number of attached ions on the surface during the test.

**Figure 7:**
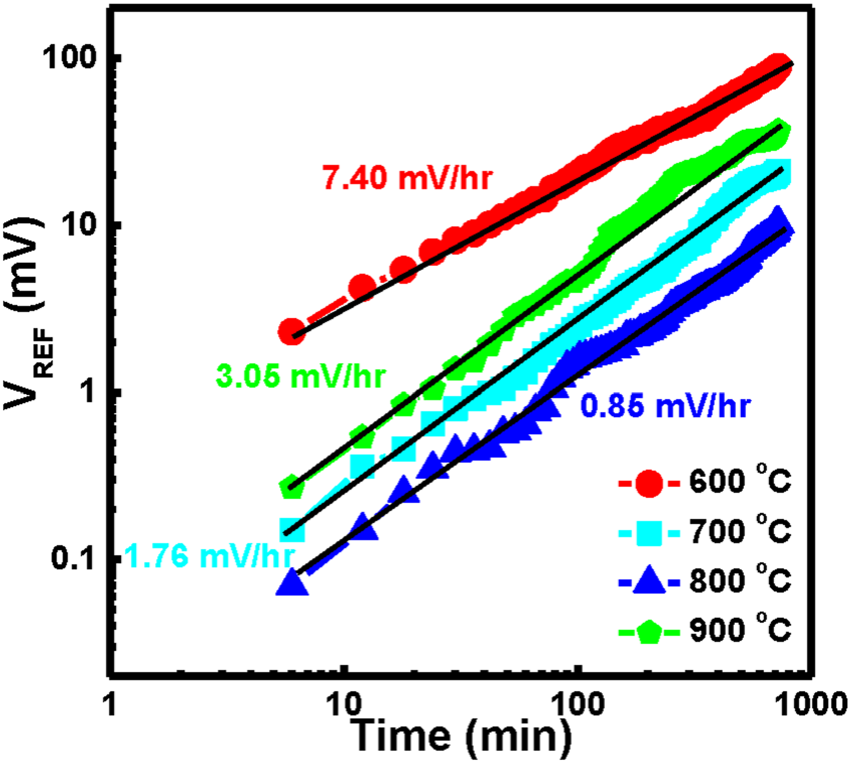
Drift rates measured in solution at pH 7 of CeY_*x*_O_*y*_ EIS sensors that had been prepared with annealing at various temperatures.

According to the physical model for gate voltage drift proposed by Jamasb *et al*.^51^, the overall capacitance of gate insulator (the series combination of the capacitance of the modified surface layer and the underlying insulator) should exhibit a slow, temporal variation. Therefore, the amount of inversion charge stored in the semiconductor at a given gate bias would slowly change over time, leading to a monotonic temporal change in the threshold voltage and, thus, in the drain current; this phenomenon is commonly known as “drift.” Chemical modification of the insulator surface can lead to a variation in the number of surface sites over time, thereby resulting in a monotonic temporal increase in the threshold voltage. Figure 7 displays the drift characteristics of the CeY_*x*_O_*y*_ EIS devices prepared with annealing at the various RTA temperatures, measured in solution at pH 7 for 12 h. Here, the change in the reference voltage (Δ*V*_REF_) is defined as

10 $${\rm{\Delta }}{V}_{REF}={V}_{REF}(t)-{V}_{REF}(0)$$

The degradation slope of the reference voltage represents the EIS stability. The drift rates of the CeY_*x*_O_*y*_ EIS devices after RTA at 600, 700, 800, and 900 °C were 7.40, 1.76, 0.85, and 3.05 mV/h, respectively. Thus, the CeY_*x*_O_*y*_ EIS device annealed at 800 °C had the best long-term stability, while the device annealed at 600 °C exhibited a serious drift rate. The drift in the reference voltage might result from lattice defects (e.g., vacancies or dangling bonds) capturing clusters of ions. We suspect that the less-pronounced drift behavior after RTA at 800 °C arose from a lower density of crystal defects; that is, these annealing conditions could effectively remove such defects. In addition, a higher drift rate may be due to a larger number of crystal defects producing buried surface sites and/or traps.

## Conclusion

We have observed a super-Nernstian response to pH from EIS devices incorporating CeY_*x*_O_*y*_ sensing membranes grown on Si substrates through reactive co-sputtering. XRD, XPS, and AFM confirmed the presence of (CeY)O_2_ structures in these EIS devices. The pH-sensing performance of the EIS device incorporating the CeY_*x*_O_*y*_ film that had been annealed at 800 °C was superior to those of the devices subjected to other annealing temperatures, including a higher sensitivity (78.15 mV/pH), a smaller hysteresis voltage (1.4 mV), and a lower drift rate (0.85 mV/h). This enhanced performance resulted from the formation of a stoichiometric (CeY)O_2_ film, a high surface roughness, and a low number of crystal defects after applying these annealing conditions. The super-Nernstian pH-response appears to have resulted from the Y ions incorporated within the Ce framework enhancing the valence change of the Ce oxidation state, thereby causing less than one electron to be transferred per proton in the redox reaction, due to the mixed oxidized and reduced CeO_*x*_ states in the solution.
